# Population-Based Survival of Childhood and Adolescent Cancers (0–19 Years) in Madrid: Analysis by Sex, Age, Tumour Type, and Stage

**DOI:** 10.3390/cancers17193113

**Published:** 2025-09-24

**Authors:** Raquel López-González, David Parra-Blázquez, Daniel Moñino, Candela Pino-Rosón, Clotilde Sevilla-Hernández, Marina Pollán, Nuria Aragonés

**Affiliations:** 1 Faculty of Medicine, Universidad Autónoma, C/Arzobispo Morcillo 4, 28029 Madrid, Spain; candela.pr@salud.madrid.org; 2 Cancer Surveillance and Registry Unit, General Directorate of Public Health, Madrid Health Department, C/López de Hoyos 35, 28002 Madrid, Spainc.sevilla.hernandez@salud.madrid.org (C.S.-H.); nuria.aragones@salud.madrid.org (N.A.); 3 Primary Care Department, Madrid Health Department, C/San Martín de Porres 6, 28035 Madrid, Spain; daniel.monino@salud.madrid.org; 4 Carlos III Health Institute, Av. de Monforte de Lemos 5, 28029 Madrid, Spain; mpollan@isciii.es; 5 Consortium for Biomedical Research in Epidemiology and Public Health (CIBERESP), Av. De Monforte de Lemos 3–5, N 11, 28029 Madrid, Spain

**Keywords:** childhood cancer epidemiology, cancer survivors, childhood cancer, adolescent cancer, survival of children with cancer, population-based cancer registry, childhood cancer registries, Toronto Paediatric Cancer Stage Guidelines

## Abstract

**Background/Objectives:** In Europe, over 80% of children diagnosed with cancer survive at least 5 years. To improve cancer monitoring, the Paediatric Population-Based Cancer Registry (PCRM) was established in the Community of Madrid. This study aimed to describe population-based 1-, 3- and 5-year survival for children and adolescents diagnosed with cancer, by sex, age, tumour type and stage at diagnosis. **Methods:** Data were extracted from the PCRM, which reviews all cases identified through integrated primary care, hospital discharge, and mortality data, using electronic medical records. Patients aged 0–19 diagnosed with primary malignant cancer between 2015 and 2018 were included, with follow-up for vital status through October 2024. Stage was classified using the 2014 Toronto Childhood Cancer Staging Guidelines (tier 2). Kaplan–Meier methods were used to estimate survival, and log-rank tests assessed group differences. Cox regression was used to quantify the effect of localized vs. advanced disease. **Results:** The analysis included 862 patients. Most frequent cancers were leukaemia (24.1%), lymphomas (22.2%) and central nervous system (CNS) tumours (12.6%). Stage was assigned to 88.4% tumours. Overall survival was 93.6% in 1 year and 85.9% in 5 years. Five-year survival was 83.7% for leukaemia, 97.4% for lymphomas, 66.1% for CNS tumours; 85.8% in boys vs. 85.9% in girls (*p* = 0.908); 85.2% in children aged 0–14 years vs. 87.8% in adolescents aged 15–19 years (*p* = 0.314); and 69.9% for advanced vs. 89.7% for early-stage (*p* < 0.001), with a 3.3-fold higher mortality risk. **Conclusions:** This population-based study offers promising survival estimates reaching 86% globally at 5 years while revealing differences by cancer type and stage. It also highlights the Toronto Guidelines as a valuable tool for standardizing cancer registry methods and providing useful epidemiological indicators.

## Simple Summary

Cancer is uncommon in children and adolescents, but it remains one of the main causes of death in these age groups. To better understand how cancer affects young people, the Community of Madrid (Spain) established a population-based cancer registry to track all cases in those under 20 years old. This study looks at recent data from 862 children and adolescents diagnosed with malignant cancer between 2015 and 2018. Most young patients—over 93%—survived at least one year after diagnosis, and nearly 86% lived for five years or more. These survival rates were similar for boys and girls, as well as between younger children and teenagers. However, the stage of cancer at diagnosis made a big difference: those with advanced cancer had a 3.3 times higher risk of dying compared to those diagnosed earlier. These findings highlight the importance of early detection and continued monitoring of cancer outcomes in young populations.

## 1. Introduction

Cancer is an uncommon disease in childhood and adolescence. In the Community of Madrid (CM), approximately 215 children aged 0 to 19 years are diagnosed with a malignant cancer each year, corresponding to an incidence rate of 166.8 cases per million person-years [1]. Although uncommon, cancer is the leading cause of death among those aged 1 to 14 years and the second leading cause among adolescents aged 15 to 19 years in Spain [2]. In Europe, 5-year age-standardized survival for all childhood cancers combined has improved significantly over time, increasing from 78% (95% CI: 78–79%) in 2004–2006 to 81% (81–82%) in 2010–2014 among children aged 0 to 14 years [3]. However, unlike many types of cancer in adulthood, most pediatric cancers cannot be prevented. Notably, childhood cancer mortality rates have decreased over recent decades, both worldwide [4] and in the CM [5].

To date, no population-based information on childhood cancer survival has been available specifically for the CM. Existing data come either from hospital-based studies within Madrid [6] or from national-level estimates [7]. However, monitoring cancer incidence and survival in children and adolescents at a population level is crucial for tracking its evolution over time and for coordinating public health strategies. Population-based cancer registries provide an unbiased view of cancer survival and allow close monitoring of each case to determine the time elapsed from diagnosis to death.

To address this gap, the CM launched the Population-Based Cancer Registry for Childhood and Adolescence of the Community of Madrid (PCRM), which contains validated information for all tumours diagnosed in the population under 20 years of age residing in the CM at the time of diagnosis. The PCRM is the first population-based cancer registry in the CM, covering approximately 1.3 million individuals under 20 years of age, and is integrated into the European Network of Cancer Registries, ensuring harmonized data collection and international comparability [8]. By including all cases diagnosed among residents, the PCRM provides a comprehensive and unbiased resource for monitoring incidence and survival.

The aim of this study was to describe, for the first time, population-based 1-, 3- and 5-year survival rates of patients aged 0–19 years with cancer in the CM, by sex, age, type of tumour and stage at diagnosis.

## 2. Materials and Methods

The data included in this analysis were extracted from the PCRM. It includes all malignancies (except non-melanoma skin cancer), non-malignant tumours of the Central Nervous System (CNS) and benign and in situ tumours of the bladder, diagnosed in people under 20 years who had resided in the CM for at least six months before diagnosis (or since birth for children under six months of age). Data collection combines passive and active case finding through cross-referencing data from primary care, hospital discharge, and mortality records, followed by manual validation of potential incident cases through retrospective review of electronic medical charts. Duplicate records are identified and resolved during the linkage process by matching several characteristics (e.g., name, surname, ID when available, date of birth, year of diagnosis, tumour morphology and topography). Any potential duplicate is manually reviewed to confirm uniqueness or identify twin siblings. The main indicators used to assess data quality (i.e., validity, completeness, and comparability), as well as further details on the registry’s methodology have been published elsewhere [1,9].

### 2.1. Study Population

The study population included individuals aged 0 to 19 years diagnosed with a malignant cancer as described above, during the entire period for which data were available in the registry (2015–2018) in the CM.

The following cases were excluded from the analysis: those identified by death certificate only, diagnosed by autopsy, with in situ neoplasms, and those with a tumour not classifiable by the International Classification of Childhood Cancer 3rd edition (ICCC-3) [10].

For clarity, throughout the manuscript we refer to “childhood cancer” as cases diagnosed at ages 0–14 years, “adolescent cancer” as cases diagnosed at ages 15–19 years, and “paediatric cancer” as encompassing both groups (<20 years).

### 2.2. Follow-Up

Cases were followed-up for vital status ascertainment until 23 October 2024. Vital status is ascertained through the National Death Index (INDEF by its acronym in Spanish), which provides the date of death without the cause. The INDEF allows automated cross-matching using an individual’s unique personal identifier (national ID, passport or foreigner identity number) or, in the absence of these (as is common among children aged less than 14 years), through combinations of surnames, given name, date of birth, and sex. For residents of the CM with a single surname (mostly foreign-born), manual cross-checking is performed using the INDEF’s refined individual search process which applies hierarchical matching levels based on different combinations of multiple identifiers (i.e., surname(s), given name, initials of first/second surname or name, date of birth, sex, ID and date of death) [11]. All potential matches are manually reviewed by PCRM staff to confirm their validity. This multi-level approach minimizes the risk of false matches and supports comprehensive vital status retrieval. Updated vital status information is subsequently incorporated into the PCRM’s database, nested in the CanReg5 web application developed by the International Agency for Research on Cancer.

### 2.3. Statistical Analysis

Statistical analysis was performed using the Kaplan–Meier method and the log-rank test to identify significant differences between groups. Individuals not identified as deceased in the INDEF were considered alive and therefore censored at the end of the follow-up period.

The analysis was stratified by sex (boy/girl), age groups (<1 year, 1–4 years, 5–9 years, 10–14 years, 15–19 years), tumour group/subgroup according to ICCC-3 [10], and tumour stage according to the tier 2 of the 2014 Toronto Childhood Cancer Staging Guidelines (henceforth referred to as the Toronto Guidelines) [12,13]. The Toronto Guidelines are an internationally developed framework that harmonizes and unifies multiple disease-specific staging systems into a common approach suitable for pediatric cancers [12]. They were specifically designed for use by population-based cancer registries to enable uniform and consistent reporting of stage at diagnosis across countries and cancer types, avoiding reliance on adult-oriented systems such as TNM, which are not applicable to most childhood malignancies. For the stratification by stage, tumours with unknown stage at diagnosis were excluded. To summarize survival by stage, two categories were created: early and advanced stage. Consistent with the definitions used by cancer surveillance agencies in Australia [14], “early stage” tumours were defined as those confined to the site of origin or to regional lymph nodes (localized, locoregional, regional, limited, stages I and II of lymphomas, and CNS-negative stage of leukaemia), while “advanced stage” tumours were those spread to other parts of the body (metastatic stages, stages III and IV of lymphomas, and MS stage of neuroblastomas). Cox regression was used to quantify the effect of these two categories, with the corresponding hazard ratio reported both unadjusted and adjusted for sex, age at diagnosis, and diagnostic group according to the ICCC-3.

All statistical analyses were conducted using Stata/IC 16.1 (StataCorp LLC, College Station, TX, USA).

### 2.4. Ethics Statement

This study was conducted in accordance with the Declaration of Helsinki and data protection regulations established by the Spanish Law 41/2002 of 14 November 2002, concerning patient autonomy, rights, and obligations regarding information and clinical documentation. Ethical review and approval were waived for this study as well as informed consent, as Article 16.3 of Law 41/2002 stipulates that informed consent is not required for accessing patients’ identifying data for epidemiological or public health reasons, provided anonymity is maintained. We present aggregate anonymized data to prevent the identification of individuals.

## 3. Results

Between 2015 and 2018, 1002 patients under 20 years were registered in the PCRM. Of these, 5 were diagnosed through the death certificate only, 4 were unclassifiable according to ICCC-3 (one of which was an in situ neoplasm), 53 were benign, and 78 had uncertain behaviour. No patients were diagnosed by autopsy, leaving 862 individuals included in the analysis. Of those, 478 (55.5%) were boys, 229 (26.6%) were between 15 and 19 years old, and the most predominant types of cancer were leukaemia (24.1%), lymphomas (22.2%) and CNS neoplasms (12.6%) (Table 1).

Out of the 862 tumours, 621 (72.0%) were stageable according to the Toronto Guidelines. The total completeness of Toronto Guidelines’ tier 2 was 88.4% (*n* = 549). Ewing sarcomas (76.5%; *n* = 26/34) and ovarian cancers (60%; *n* = 6/10) were the only tumour types with less than 80% completeness, while retinoblastomas (100%; *n* = 20/20) and ependymomas (100%; *n* = 13/13) demonstrated the highest percentage of staged cases (Table 2). More than three quarters (81.2%; *n* = 446) of the children had early-stage cancers. This distribution varied widely by type of cancer.

Of the 862 children, 139 had died by October 2024 (75 boys and 64 girls). The mortality rate was 3.14 per 100 patients over a total of 3 890.16 person-days at risk.

**Figure 1:**
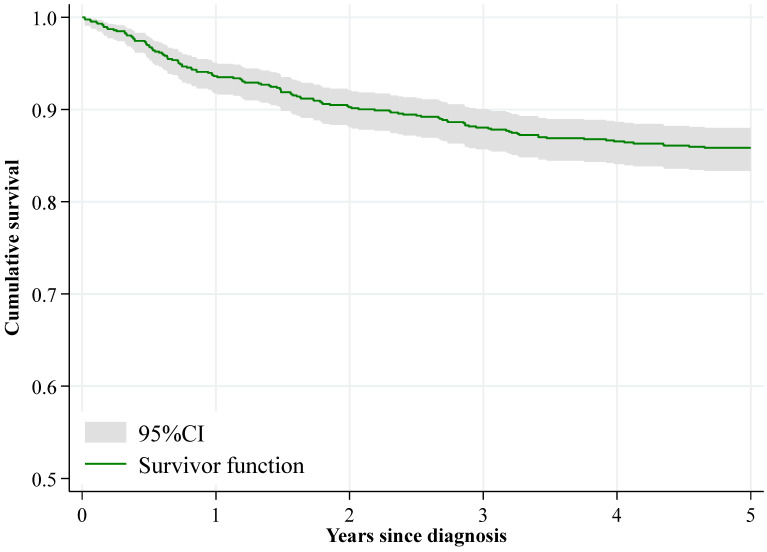
Kaplan–Meier observed survival curves (with 95% confidence interval) for all malignant incident tumours, both sexes. Community of Madrid, 2015–2018.

The overall 1-year survival was 93.6%, while 5-year survival decreased to 85.9% (Figure 1, Table 3). There were no differences in survival by sex (*p* = 0.908). The survival probability declined over follow-up for all age groups, with a more pronounced decrease in children aged 10–14 years (*p* = 0.002), who exhibited the lowest survival at 5 years among all age groups: 77.4%. Children younger than 1 year of age experienced a rapid decline in survival probability during the first year after diagnosis (88.9%), but this trend stabilized over time. There were no significant differences in survival between patients aged 0–14 years and 15–19 years (*p* = 0.314).

Leukaemia exhibited a consistent decline in survival throughout the follow-up period, dropping from 90.9% in the first year to 83.7% at 5 years. Conversely, survival rates for lymphomas remained highly stable throughout the follow-up, with 1-, 3- and 5-year survival close to 100%. CNS neoplasms presented the lowest survival estimate among all types of tumours at any time point of follow-up, declining from 86.2% in the first year to 66.1% at 5 years. Retinoblastomas and renal tumours stood out with survival estimates of 100% at 5 years. Malignant bone tumours exhibited a decline in survival from 94.6% at 1 year one to 77.0% by 5 years. In contrast, soft tissue and other extraosseous sarcomas showed a more gradual decline, from 86.5% at 1 year to 82.7% at 5 years. Germ cell tumours had relatively high survival rates across follow-up (96.3% to 94.4%), similarly to epithelial neoplasms and melanomas (95.4% to 90.8%) (Table 3).

**Figure 2:**
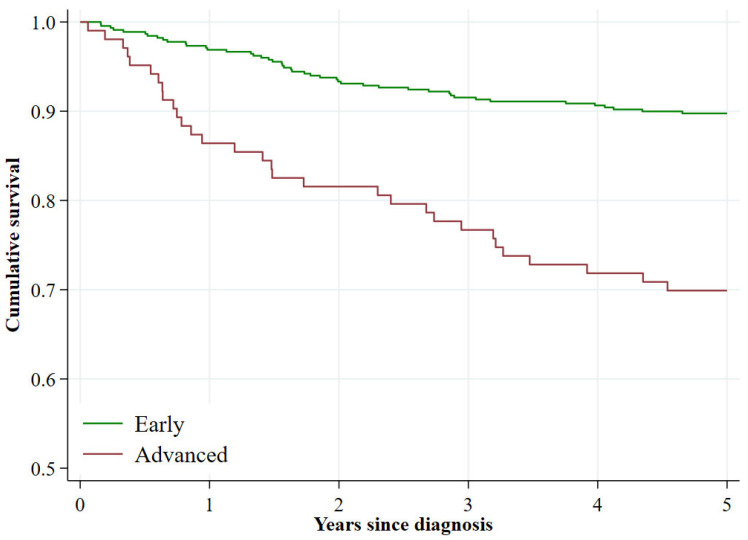
Kaplan–Meier observed survival curves for all incident malignant tumours by two stage categories, both sexes. Community of Madrid, 2015, 2018.

Children diagnosed at advanced stages of the disease had significantly lower survival estimates than those diagnosed at early stages with 5-year survival estimates of 69.9% and 89.7%, respectively (*p* < 0.001) (Figure 2, Table 3). According to the Cox regression analysis, individuals diagnosed at advanced stages had up to 3.3 times higher risk of death (95% CI 2.1–5.2). This risk increased to 4.0 after adjusting for sex, age at diagnosis, and diagnostic group according to the ICCC-3.

**Figure 3:**
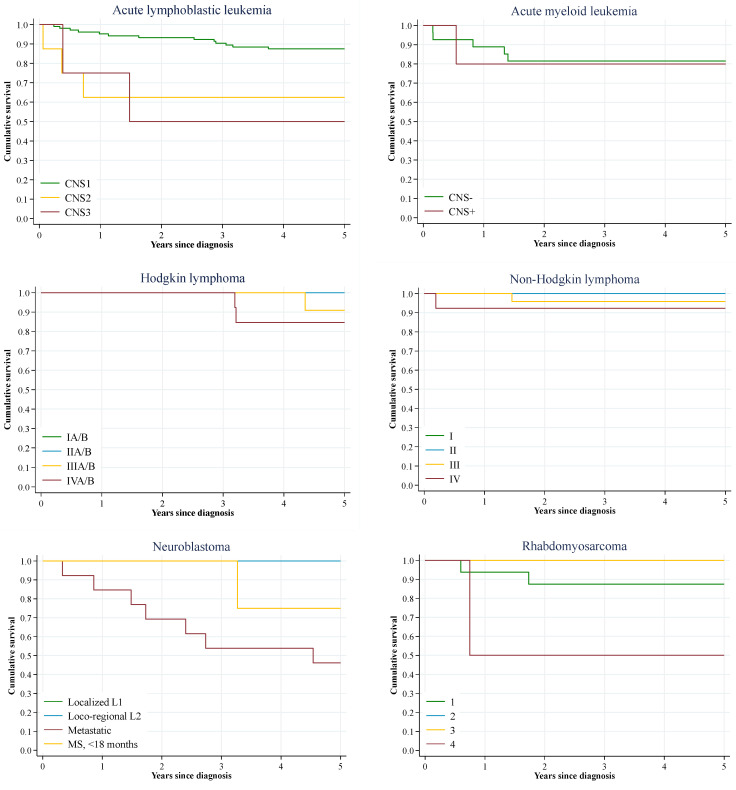

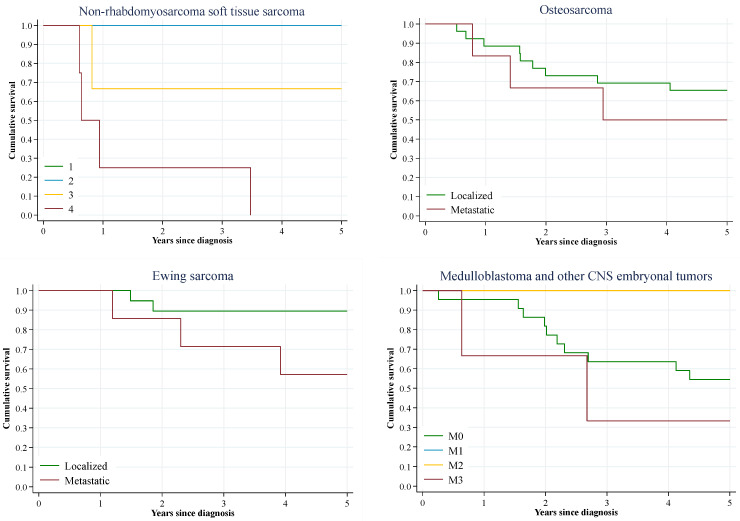
Kaplan–Meier observed survival curves for all incident malignant tumours, by type of cancer and stage at diagnosis. Community of Madrid, 2015, 2018.

Figure 3 displays the Kaplan–Meier curves by stage for tumours included in the Toronto Guidelines. The largest differences in 5-year observed survival were seen in acute lymphoblastic leukemia (87.5% for CNS1 vs. 62.5% for CNS2 and 50.0% for CNS3; *p* = 0.007), Hodgkin lymphoma (100% for stage I–II vs. 84.6% for stage IV; *p* = 0.045), neuroblastoma (100% for stage I–II vs. 46.2% for stage IV and 75.0% for metastatic cases <18 months old; *p* = 0.002), and non-rhabdomyosarcoma soft tissue sarcoma (100% for stage I-II vs. 66.7% for stage III, with a 3-year survival of 25.0% for stage IV and no cases at risk for 5-year survival; *p* < 0.001). The specific survival estimates by the stage at diagnosis are available in Table S1.

## 4. Discussion

This population-based study provides a comprehensive analysis of childhood and adolescent cancer survival based on key factors including sex, age, tumour type, and stage at diagnosis.

The overall 5-year survival for all malignant neoplasms was 85.9% (95% CI: 83.3–88.0), slightly higher than the estimate for Spain based on a pooled analysis of 11 Spanish cancer registries participating in the EUROCARE-6 project (81.1%) and higher than the estimate for 31 European countries (81.3%) [3]. This higher survival estimate was particularly evident in acute myeloid leukaemia (81.3% vs. 66.7% in Spain and 70.4% across Europe), non-Hodgkin lymphomas (96.8% vs. 89.8% and 87.9%), CNS neoplasms (66.1% vs. 57.9% and 59.4% e), Ewing sarcomas (80.8% vs. 69.2% and 70.3%), and rhabdomyosarcomas (87.0% vs. 73.1% and 71.0%), respectively. These differences may be mainly explained by two factors: first, the EUROCARE-6 project included only children aged 0–14 years, whereas our analysis included adolescents aged 15–19, who tend to have higher survival. Second, our study covered more recent diagnoses (2015–2018), while EUROCARE-6 covered diagnoses between 2000 and 2013 [3]. Recent advancements in cancer treatments may also contribute to these discrepancies, such as molecular profiling [15], and improved management of long-term treatment effects [16]. However, our results should be interpreted with caution, as we analyzed 862 cases vs. 1 898 from Spain and 35 365 across Europe [3]. Future research with larger sample sizes and longer follow-up would help clarify whether these differences reflect genuine survival improvements or are due to chance. Furthermore, while cancer registry methods are highly standardized, continued refinements in data collection and coding procedures remain key to ensure that regional and international comparisons are not affected by variations in tumour classification or coding practices.

This study found no significant differences in survival by sex, consistent with EUROCARE-6 results [3]. Since children are still in a developmental stage, sex hormones are not considered significant risk factors, as reflected in similar incidence rates –in the CM, the male-to-female ratio during 2015–2018 was 1.1 [1]—and, consequently, in comparable survival estimates. Regarding potential environmental or exogenous risk factors, boys and girls generally share similar living environments and lifestyle habits. Therefore, if significant sex-based mortality differences were observed, gender-related disparities should be further investigated [17].

Overall survival differed significantly between specific age groups (<1, 1–4, 5–9, 10–14, 15–19 years). In general, all age groups had survival above 84%, except for those aged 10–14 years (77.4%). This may be attributed to the fact that nearly half of bone malignancies are diagnosed at this age [1], and these have some of the poorest survival estimates, second only to malignant CNS tumours. Despite these age-related differences, we did not find any significant differences in survival between childhood (0–14 years) and adolescence (15–19 years). However, this should be interpreted with caution, as we are comparing survival estimates globally rather than differences within specific tumour types. Historically, adolescents and young adults (AYA) have experienced smaller survival gains compared to other age groups, a phenomenon known as the “AYA gap” [18]. One contributing factor was the better outcomes observed when AYA patients were treated in pediatric units using pediatric protocols, rather than in adult oncology settings [18,19], although more recent studies suggest that survival outcomes are now comparable between adolescents treated at adult and pediatric centres [20,21]. According to the Adolescent Cancer Working Group of the Spanish Society of Pediatric Hematology and Oncology, the proportion of patients up to 18 years of age treated in pediatric units in Spain has increased from 35.9% to 77.5% over the past decade, alongside the development of specialized multidisciplinary units for adolescent cancer care [22], which has improved the management of these patients. Furthermore, in universal healthcare systems such as Spain’s, access to diagnostics, treatment, and follow-up care is generally equitable across age groups, which may help minimize age-related disparities in survival. While adolescents have a distinct cancer profile compared to younger children, survival studies in this group remain limited, as many pediatric cancer registries do not include patients aged 15–19 years [23]. When comparing the 5-year survival of adolescents in the CM (87.8%; 95% CI: 82.8–91.4) with published data from other high-income regions, such as the United States or Korea [24,25], our findings fall within the previously reported ranges.

The results by tumour group show that the lowest survival was found among individuals diagnosed with malignant CNS tumours, with a 5-year survival of 66.1% (95% CI: 56.4–74.1) aligning with other European countries [3,26]. While this may seem low compared to other tumour groups, it is slightly higher than the estimated survival for Spain (57.9%) and Europe (59.4%) [3]. However, it is unclear if this variation in survival between regions is real or caused by different methodology among registries including changes in inclusion criteria (e.g., inclusion of benign/uncertain/malignant neoplasms), coding practices and variation in registration of tumours without microscopic verification, or completeness of follow-up [27]. Poorer survival in malignant CNS tumours can be attributed to several factors. The most important is that these tumours are biologically heterogeneous [28] and often located in surgically challenging or functionally critical areas, which limits the feasibility of complete resection and affects survival as well as short- and long-term quality of life. Furthermore, the most common symptoms (headache, vomiting, lethargy, and seizures) are highly nonspecific and could be misattributed to benign conditions [29], which have more favourable survival outcomes [30]. This can lead to delays in diagnosis, allowing tumour growth, complicating surgical resection, and ultimately worsening prognosis. Pathologists and clinicians also face challenges in accurately classifying CNS tumours due to their complex morphology and behaviour. This is further complicated by changes in tumour classification over time, as reflected in successive versions of the ICD-O, where some tumours previously considered malignant have been reclassified as non-malignant (e.g., pilocytic astrocytoma), while others have been newly designated as malignant (e.g., papillary meningioma).

When comparing Madrid to other regions, disparities in survival estimates become particularly notable, especially between high-income regions and areas such as Sub-Saharan Africa [31,32]. For instance, 5-year survival in Rwanda has been reported as 29.8% for lymphoid leukaemia compared to 84.5% in the CM, for retinoblastomas 68.0% vs. 100%, for osteosarcomas 34.5% vs. 62.5%, and for rhabdomyosarcomas 29.8% vs. 87.0% [31]. These differences can be attributed to several factors, including limited access to early diagnostic tools, inadequate healthcare infrastructure, and a lack of specialized treatment options [33,34,35].

Reporting stage, tumour size, and spread extent for pediatric patients has historically been less straightforward for pediatric patients [36,37]. Before the Toronto Guidelines, data on stage of childhood cancers came from clinical trials where patient eligibility criteria and varying study conditions introduced significant heterogeneity limiting generalizability. The Toronto Guidelines provide a standardized framework that enhances consistency across cancer registries. The ongoing BENCHISTA multicenter project is leveraging these guidelines to analyze survival by stage [38,39,40], with the PCRM actively contributing data to this international initiative. The completeness of the Toronto Guidelines was 88.4% which falls within the range reported in other studies (67.3–96%) [26,31,32,38,41,42,43,44,45,46,47,48]. A limited number of population-based studies have reported survival by stage at diagnosis using the Toronto Guidelines, including studies from a group of European countries [26], Italy [44], Australia [45,46,47], Brazil [48] and Sub-Saharan Africa [31,32,43].

Survival estimates by stage at diagnosis in the CM are generally in line with those observed in European countries and Australia [26,44,45,46,47]. Minor differences may reflect variation in staging accuracy between registries, though they are more likely due to differences in sample size. For instance, survival for M0-stage medulloblastoma in the CM was 54.6%, compared to 75% in Italy [44], but these estimates were based on just 12 patients in the CM versus 31 in Italy. Similarly, 5-year survival for metastatic neuroblastoma in the CM was 46.2%, while Australian studies reported 60.0–61.6% [45,46], a difference likely explained by the smaller number of cases in Madrid (*n* = 6) versus approximately 190 in the Australian studies. Nevertheless, it is possible that other factors contribute to the observed differences, such as variations in treatment protocols, clinical practices, access to specialized care, and tumour classification. Studies with larger sample sizes and further investigation into these variables will be necessary to better understand the underlying causes.

Conversely, survival figures were consistently higher in the CM than in Mato Grosso, Brazil [48] and in Sub-Saharan Africa [31,32,43], underscoring again the disparities between high-income and low- and middle-income countries. While both Spain and Brazil have universal healthcare systems, Brazil faces significant regional inequalities, with certain areas having limited access to specialized healthcare, particularly in rural regions or economically disadvantaged communities [49]. The differences with Sub-Saharan Africa were particularly striking for retinoblastomas. In the CM, there were no cases of stage IV retinoblastoma among 20 diagnosed patients, similar to findings in Australia [41,46] and several European countries [26]. In contrast, Sub-Saharan African countries reported metastatic cases in 19–42.5% of retinoblastoma patients [31,32,43], which significantly impacts overall survival estimates. For example, 1-year survival estimates ranged from 60 to 77.8% in Sub-Saharan Africa [31,32,43] compared to 100% in Madrid. This disparity can likely be explained by the shift toward eye-conserving treatments in high-income regions, enabled by advancements in therapy [45,50].

This study has several strengths. It provides population-level coverage of all childhood and adolescent cancer patients diagnosed in the CM during the study period covering a population of 1.3 million inhabitants younger than 20 years of age. Furthermore, it is among the few studies to use the Toronto Guidelines for survival analysis by stage at diagnosis. It is also among the most extensive studies in this field, as survival data are often reported separately for hematological and solid tumours or analyzed independently by stage or demographic characteristics. Additionally, it is one of the few studies to include adolescents aged 15–19, offering a comprehensive analysis of this age group cancer survival estimates.

There are also some limitations to consider. This study utilized the 2014 version of the Toronto Guidelines, which were updated in 2019 [13]. Additionally, stage at diagnosis was recorded by a single reviewer, raising a potential risk of misclassification, although the risk is expected to be minimal as most medical charts explicitly stated the stage as determined by the treating oncologist who had full access to full diagnostic workups, and because complex or ambiguous diagnoses were reviewed by at least three staff members of the PCRM. The use of the Toronto Pediatric Cancer Stage Guidelines adds value by enabling standardized staging and comparability across studies. However, applying these guidelines retrospectively can be challenging, particularly when clinical information is incomplete or inconsistently documented. In our study, staging information was available for 88.4% of cases, a level of completeness that might limit the representativeness of our estimates at stage level. However, the percentage of unstaged tumours is consistent with other published studies, suggesting that this limitation is unlikely to compromise the validity of the results. Another limitation is that, although we present stratified results by key demographic and diagnostic variables (sex, age at diagnosis, stage and tumour type), we did not have information on other potential confounders, such as treatment modality, socioeconomic deprivation, underlying cancer predisposition syndromes, or ethnic disparities. Future research would benefit from incorporating these variables into the analysis to provide a more comprehensive understanding of survival in childhood and adolescent cancer. Lastly, while the study includes a big cohort diagnosed over a four-year period, this relatively short timeframe for such infrequent diseases as childhood tumours limits the number of cases and, consequently, the precision of survival estimates. This also affects some stage-specific analyses, where small numbers in certain categories reduce statistical power, although staging was reported according to the internationally accepted Toronto Guidelines to ensure comparability.

## 5. Conclusions

In conclusion, this study reports a population-based 5-year survival estimate of 85.9% (95% CI: 83.3–88.0) for childhood and adolescent cancer (<20 years of age), with no significant differences by sex but lower survival among patients aged 10–14 years, possibly related to a higher incidence of bone tumours. Importantly, our findings highlight stage at diagnosis as a key prognostic determinant of survival outcomes in childhood and adolescent cancer, with individuals diagnosed at advanced stages having a 3.3-fold higher risk of death. This study reinforces the value of the Toronto Childhood Cancer Staging Guidelines as a robust tool for standardizing survival analysis by stage at diagnosis among cancer registries. These results mark an important step towards providing reliable and consistent survival data for childhood and adolescent cancer and further underline the role of population-based cancer registries as essential tools for monitoring cancer.

## Figures and Tables

**Table 1:** Characteristics of the cohort by sex, age and type of cancer. Community of Madrid, 2015–2018.

	*n*	%
Sex		
Boys	478	55.5
Girls	384	44.6
Age at diagnosis		
<1 years	63	7.3
01–04 years	193	22.4
05–09 years	187	21.7
10–14 years	190	22.0
15–19 years	229	26.6
Type of cancer according to ICCC-3		
I. Leukaemias, Myeloproliferative and Myelodysplastic Diseases	208	24.1
II. Lymphomas and reticuloendothelial neoplasms	191	22.2
III. CNS and Miscellaneous Intracranial and Intraspinal Neoplasms	109	12.6
IV. Neuroblastoma and Other Peripheral Nervous Cell Tumours	43	5.0
V. Retinoblastoma	20	2.3
VI. Renal Tumours	33	3.8
VII. Hepatic Tumours	9	1.0
VIII. Malignant Bone Tumours	74	8.6
IX. Soft Tissue and Other Extraosseous Sarcomas	52	6.0
X. Germ Cell Tumours, Trophoblastic Tumours and Neoplasms of Gonads	54	6.3
XI. Other Malignant Epithelial Neoplasms and Malignant Melanomas	65	7.5
XII. Other and Unspecified Malignant Neoplasms	4	0.5
All	862	100.0

**Table 2:** Distribution of incident tumour types by stage at diagnosis according to the Toronto Childhood Cancer Stage Guidelines, tier 2. Community of Madrid, 2015–2018.

Type of Tumour	Staging Category				
Acute lymphoblastic leukemia (*n* = 116; 18 missing)	CNS1	CNS2	CNS3		
*n*	104	8	4		
%	89.7	6.9	3.4		
Acute myeloid leukemia (*n* = 32; 7 missing)	CNS-	CNS+			
*n*	27	5			
%	84.4	15.6			
Hodgkin lymphoma (*n* = 79; 3 missing)	IA/IB	IIA/IIB	IIIA/IIIB	IVA/IVB	
*n*	12	43	11	13	
%	15.2	54.4	13.9	16.5	
Non-Hodgkin lymphoma (*n* = 63; 4 missing)	I	II	III	IV	
*n*	7	19	24	13	
%	11.1	30.2	38.1	20.6	
Neuroblastoma (*n* = 38; 5 missing)	L1	L2	MS	M	
*n*	13	8	4	13	
%	34.2	21.1	10.5	34.2	
Wilms tumour (*n* = 27; 5 missing)	I/y-I	II/y-II	III/y-III	IV	
*n*	14	6	3	4	
%	51.9	22.2	11.1	14.8	
Rhabdomyosarcoma (*n* = 23; 4 missing)	I	II	III	IV	
*n*	16	2	3	2	
%	69.6	8.7	13.0	8.7	
Non-rhabdomyosarcoma (*n* = 20; 4 missing)	I	II	III	IV	
*n*	10	3	3	4	
%	50.0	15.0	15.0	20.0	
Osteosarcoma (*n* = 32; 4 missing)	Localized	Metastatic			
*n*	26	6			
%	81.3	18.8			
Ewing sarcoma (*n* = 26; 8 missing)	Localized	Metastatic			
*n*	19	7			
%	73.1	26.9			
Retinoblastoma (*n* = 20; 0 missing)	0	I	II	III	IV
*n*	11	7	-	2	-
%	55.0	35.0	-	10.0	-
Hepatoblastoma (*n* = 6; 1 missing)	Localized	Metastatic			
*n*	6	-			
%	100.0	-			
Testicular cancer (*n* = 20; 2 missing)	I	II	III		
*n*	12	5	3		
%	60.0	25.0	15.0		
Ovarian cancer (*n* = 6: 4 missing)	I	II	III	IV	
*n*	5	1	-	-	
%	83.3	16.7	-	-	
Medulloblastoma (*n* = 28; 3 missing)	M0	M1	M2	M3	M4
*n*	22	2	1	3	-
%	78.6	7.1	3.6	10.7	-
Ependymoma (*n* = 13; 0 missing)	M0	M1	M2	M3	M4
*n*	13	-	-	-	-
%	100.0	-	-	-	-

**Table 3:** Observed survival at 1, 3, and 5 years by sex, age, type of tumour and stage at diagnosis. Community of Madrid, 2015–2018.

Characteristic	OS at 1 Year		OS at 3 Years		OS at 5 Years		Log, Rank Test *p*
	*n* at Risk	% (CI)	*n* at Risk	% (CI)	*n* at Risk	% (CI)	
By sex							0.908
Boys	445	92.9 (90.2, 94.9)	420	87.7 (84.4, 90.3)	410	85.8 (82.3, 88.6)	
Girls	364	94.5 (91.7, 96.4)	341	88.5 (84.9, 91.3)	330	85.9 (82.0, 89.0)	
By age							0.002
<1 years	57	88.9 (78.1, 94.5)	55	85.7 (74.3, 92.3)	53	84.1 (72.5, 91.1)	
01–04 years	186	95.9 (91.9, 97.9)	175	90.2 (85.0, 93.6)	171	88.6 (83.2, 92.3)	
05–09 years	180	95.7 (91.6, 97.8)	173	92.0 (87.1, 95.1)	168	89.8 (84.5, 93.4)	
10–14 years	172	90.0 (84.8, 93.5)	152	79.5 (73.0, 84.6)	147	77.4 (70.7, 82.7)	
15–19 years	217	94.3 (90.4, 96.7)	209	90.8 (86.3, 93.9)	201	87.8 (82.8, 91.4)	
By two categories of age							0.314
Childhood (0–14 years)	592	93.4 (91.1, 95.1)	552	87.1 (84.2, 89.4)	539	85.2 (82.1, 87.7)	
Adolescence (15–19 years)	217	94.3 (90.4, 96.7)	209	90.8 (86.3, 93.9)	201	87.8 (82.8, 91.4)	
By type of tumour							<0.001
I. Leukaemias, Myeloproliferative and Myelodysplastic Diseases	189	90.9 (86.1, 94.1)	178	85.6 (80.0, 89.7)	174	83.7 (77.9, 88.0)	
II. Lymphomas and reticuloendothelial neoplasms	190	99.5 (96.3, 99.9)	189	99.0 (95.9, 99.7)	186	97.4 (93.8, 98.9)	
III. CNS and Miscellaneous Intracranial and Intraspinal Neoplasms	94	86.2 (78.2, 91.5)	77	70.6 (61.1, 78.2)	72	66.1 (56.4, 74.1)	
IV. Neuroblastoma and Other Peripheral Nervous Cell Tumours	40	93.0 (79.9, 97.7)	36	83.7 (68.9, 91.9)	34	79.1 (63.6, 88.5)	
V. Retinoblastoma	20	100 (-)	20	100 (-)	20	100 (-)	
VI. Renal Tumours	33	100 (-)	33	100 (-)	33	100 (-)	
VII. Hepatic Tumours	8	88.9 (43.3, 98.4)	8	88.9 (43.3, 98.4)	7	77.8 (36.5, 93.9)	
VIII. Malignant Bone Tumours	70	94.6 (86.2, 97.9)	59	79.7 (68.7, 87.3)	57	77.0 (65.7, 85.0)	
IX. Soft Tissue and Other Extraosseous Sarcomas	45	86.5 (73.8, 93.3)	44	84.6 (71.6, 92.0)	43	82.7 (69.4, 90.6)	
X. Germ Cell Tumours, Trophoblastic Tumours and Neoplasms of Gonads	52	96.3 (86.0, 99.1)	51	94.4 (83.8, 98.2)	51	94.4 (83.8, 98.2)	
XI. Other Malignant Epithelial Neoplasms and Malignant Melanomas	62	95.4 (86.4, 98.5)	60	92.3 (82.5, 96.7)	59	90.8 (80.6, 95.7)	
XII. Other and Unspecified Malignant Neoplasms	4	100 (-)	4	100 (-)	4	100 (-)	
By two categories of stage at diagnosis ^1^							<0.001
Early stage	433	96.9 (94.8, 98.1)	409	91.5 (88.5, 93.7)	401	89.7 (86.5, 93.0)	
Advanced stage	89	86.4 (78.1, 91.7)	79	76.7 (67.3, 83.7)	72	69.9 (60.0, 77.8)	
All	807	93.6 (91.8, 95.1)	759	88.1 (85.7, 90.0)	740	85.9 (83.3, 88.0)	-

^1^ Patients with unknown stage at diagnosis were excluded; OS: Overall survival; CI: Confidence interval.

## Data Availability

The data used in this study are available upon request from the Paediatric Population-Based Cancer Registry of the Community of Madrid at the following email address: registro.cancer@salud.madrid.org.

## Supplementary Materials

The following supporting information can be downloaded at: https://www.mdpi.com/article/10.3390/cancers17193113/s1, Table S1: Observed survival at 1, 3, and 5 years by stage at diagnosis of the Toronto Guidelines. Community of Madrid, 2015, 2018.

## Abbreviations

The following abbreviations are used in this manuscript:

AYA	Adolescent and young adult
CM	Community of Madrid
CNS	Central Nervous System
ICCC-3	International Classification of Childhood Cancer 3rd edition
INDEF	National Death Index (by its acronym in Spanish)
PCRM	Population-Based Cancer Registry for Childhood and Adolescence of the Community of Madrid
