# Supplementary material for: Population-Based Survival of Childhood and Adolescent Cancers (0–19 Years) in Madrid: Analysis by Sex, Age, Tumour Type, and Stage
Source: Cancers (Basel). 2025 Sep 24;17(19):3113. doi: 10.3390/cancers17193113 (PMC12523596; doi:10.3390/cancers17193113)
Supplement: Supplementary file 1 [file cancers-17-03113-s001.zip › cancers-3817200-supplementary.pdf]

Supplementary Materials

Table S1. Observed survival at 1, 3, and 5 years by stage at diagnosis of the Toronto Guidelines. Community of Madrid, 2015, 2018.

Characteristic	OS at 1 year		OS at 3 years		OS at 5 years		Log, rank test p
	N at risk	% (CI)	N at risk	% (CI)	N at risk	% (CI)	
By stage at diagnosis[1]							<0.001
Acute lymphoblastic leukaemia	107	92.2 (85.6, 95.9)	101	87.1 (79.5, 92.0)	98	84.5 (76.5, 89.9)	0.007
CNS1	99	95.2 (88.8, 98.0)	94	90.4 (82.9, 94.7)	91	87.5 (79.5, 92.5)	
CNS2	5	62.5 (22.9, 86.1)	5	62.5 (22.9, 86.1)	5	62.5 (22.9, 86.1)	
CNS3	3	75.0 (12.8, 96.1)	2	50.0 (05.8, 84.5)	2	50.0 (05.8, 84.5)	
Acute myeloid leukaemia	28	87.5 (70.0, 95.1)	26	81.3 (63.0, 91.1)	26	81.3 (63.0, 91.1)	0.921
CNS,	24	88.9 (69.4, 96.3)	22	81.5 (07.5, 91.8)	22	81.5 (07.5, 91.8)	
CNS+	4	80.0 (17.9, 96.9)	4	80.0 (17.9, 96.9)	4	80.0 (17.9, 96.9)	
Hodgkin lymphoma	79	100 (-)	79	100 (-)	76	96.2 (88.7, 98.8)	0.045
Ann Arbor stage IA/B	12	100 (-)	12	100 (-)	12	100 (-)	
Ann Arbor stage IIA/B	43	100 (-)	43	100 (-)	43	100 (-)	
Ann Arbor stage IIIA/B	11	100 (-)	11	100 (-)	10	90.9 (50.8, 98.7)	
Ann Arbor stage IVA/B	13	100 (-)	13	100 (-)	11	84.6 (51.2, 95.9)	
Non, Hodgkin lymphoma	62	98.4 (89.3, 99.8)	61	96.8 (87.9, 99.2)	61	96.8 (87.9, 99.2)	0.614
St. Jude/Murphy stage I	7	100 (-)	7	100 (-)	7	100 (-)	
St. Jude/Murphy stage II	19	100 (-)	19	100 (-)	19	100 (-)	
St. Jude/Murphy stage III	24	100 (-)	23	95.8 (73.9, 99.4)	23	95.8 (73.9, 99.4)	
St. Jude/Murphy stage IV	12	92.3 (56.6, 98.9)	12	92.3 (56.6, 98.9)	12	92.3 (56.6, 98.9)	
Neuroblastoma	36	94.7 (80.6, 98.7)	32	84.2 (68.2, 92.6)	30	79 (62.3, 88.9)	0.002
INRGSS, localized L1	13	100 (-)	13	100 (-)	13	100 (-)	
INRGSS, loco, regional L2	8	100 (-)	8	100 (-)	8	100 (-)	
INRGSS, metastatic M	11	84.6 (51.2, 95.9)	7	53.9 (24.8, 76.0)	6	46.2 (19.2, 69.6)	
INRGSS, MS	4	100 (-)	4	100 (-)	3	75.0 (12.8, 96.1)	
Wilms tumour	27	100 (-)	27	100 (-)	27	100 (-)	NA

Characteristic	OS at 1 year		OS at 3 years		OS at 5 years		Log, rank test p
	N at risk	% (CI)	N at risk	% (CI)	N at risk	% (CI)	
Stage I/y, stage I	14	100 (-)	14	100 (-)	14	100 (-)	
Stage II/y, stage II	4	100 (-)	4	100 (-)	4	100 (-)	
Stage III/y, stage III	6	100 (-)	6	100 (-)	6	100 (-)	
Stage IV	3	100 (-)	3	100 (-)	3	100 (-)	
Rhabdomyosarcoma	21	91.3 (69.5, 97.8)	20	87.0 (64.8, 95.6)	20	87.0 (64.8, 95.6)	0.339
TNM stage 1	15	93.8 (63.2, 99.1)	14	87.5 (58.6, 96.7)	14	87.5 (58.6, 96.7)	
TNM stage 2	2	100 (-)	2	100 (-)	2	100 (-)	
TNM stage 3	3	100 (-)	3	100 (-)	3	100 (-)	
TNM stage 4	1	50.0 (06.0, 91.0)	1	50.0 (06.0, 91.0)	1	50.0 (06.0, 91.0)	
Non, rhabdomyosarcoma soft tissue sarcoma	16	80.0 (55.1, 92.0)	16	80.0 (55.1, 92.0)	15	75.0 (50.0, 88.8)	<0.001
TNM stage 1	10	100 (-)	10	100 (-)	10	100 (-)	
TNM stage 2	3	100 (-)	3	100 (-)	3	100 (-)	
TNM stage 3	2	66.7 (05.4, 94.5)	2	66.7 (05.4, 94.5)	2	66.7 (05.4, 94.5)	
TNM stage 4	1	25.0 (00.9, 66.5)	1	25.0 (00.9, 66.5)	0	NA	
Osteosarcoma	28	87.5 (70.0, 95.1)	21	65.6 (46.6, 79.3)	20	62.5 (43.5, 76.7)	0.471
Localized	23	88.5 (68.4, 96.1)	18	69.2 (47.8, 83.3)	17	65.4 (44.0, 80.3)	
Metastatic	5	83.3 (27.3, 97.5)	3	50.0 (11.1, 80.4)	3	50.0 (11.1, 80.4)	
Ewing Sarcoma	26	100 (-)	22	84.6 (64.0, 93.9)	21	80.8 (59.8, 91.5)	0.070
Localized	19	100 (-)	17	89.5 (64.1, 97.3)	17	89.5 (64.1, 97.3)	
Metastatic	7	100 (-)	5	71.4 (25.8, 92.0)	4	57.1 (17.2, 83.7)	
Retinoblastoma	20	100 (-)	20	100 (-)	20	100 (-)	NA
IRSS Stage 0	11	100 (-)	11	100 (-)	11	100 (-)	
IRSS Stage I	7	100 (-)	7	100 (-)	7	100 (-)	
IRSS Stage II	0	-	0	-	0	-	
IRSS Stage III	2	100 (-)	2	100 (-)	2	100 (-)	
IRSS Stage IV	0	-	0	-	0	-	
Hepatoblastoma	6	100 (-)	6	100 (-)	5	83.3 (27.3, 97.5)	-
Localized	6	100 (-)	6	100 (-)	5	83.3 (27.3, 97.5)	

Characteristic	OS at 1 year		OS at 3 years		OS at 5 years		Log, rank test p
	N at risk	% (CI)	N at risk	% (CI)	N at risk	% (CI)	
Metastatic	0	-	0	-	0	-	
Testicular cancer	20	100 (-)	20	100 (-)	20	100 (-)	NA
TNM stage I	12	100 (-)	12	100 (-)	12	100 (-)	
TNM stage II	5	100 (-)	5	100 (-)	5	100 (-)	
TNM stage III	3	100 (-)	3	100 (-)	3	100 (-)	
Ovarian cancer	6	100 (-)	6	100 (-)	6	100 (-)	NA
FIGO Stage I	5	100 (-)	5	100 (-)	5	100 (-)	
FIGO Stage II	1	100 (-)	1	100 (-)	1	100 (-)	
FIGO Stage III	0	-	0	-	0	-	
FIGO Stage IV	0	-	0	-	0	-	
Medulloblastoma and other CNS embryonal tumours	26	92.9 (74.4, 98.2)	18	64.3 (43.8, 78.9)	16	57.1 (37.1, 73.0)	0.440
M0	21	95.5 (71.9, 99.4)	14	63.6 (10.3, 40.3)	12	54.6 (40.3, 79.9)	
M1	2	100 (-)	2	100 (-)	2	100 (-)	
M2	1	100 (-)	1	100 (-)	1	100 (-)	
M3	2	66.7 (05.4, 94.5)	1	33.3 (09.0, 77.4)	1	33.3 (09.0, 77.4)	
M4	0	-	0	-	0	-	
Ependymoma	13	100 (-)	12	92.3 (56.6, 98.9)	11	84.6 (51.2, 95.9)	-
M0	13	100 (-)	12	92.3 (56.6, 98.9)	11	84.6 (51.2, 95.9)	
M1	0	-	0	-	0	-	
M2	0	-	0	-	0	-	
M3	0	-	0	-	0	-	
M4	0	-	0	-	0	-	
All	807	93.6 (91.8, 95.1)	759	88.1 (85.7, 90.0)	740	85.9 (83.3, 88.0)	-

[1]Patients with unknown stage at diagnosis were excluded

OS: Overall survival; NA: not applicable (test no performed because there are no failures)
